# Biomarkers of oxidant stress, insulin sensitivity and endothelial activation in rheumatoid arthritis: a cross-sectional study of their association with accelerated atherosclerosis

**DOI:** 10.1186/1756-0500-2-83

**Published:** 2009-05-09

**Authors:** Philip W Pemberton, Yasmeen Ahmad, Helena Bodill, Daniel Lokko, Samantha L Hider, Allen P Yates, Michael G Walker, Ian Laing, Ian N Bruce

**Affiliations:** 1 Clinical Research Department, Central Manchester Foundation Trust, Oxford Road, Manchester M13 9WL, UK; 2 The Kellgren Centre for Rheumatology, Central Manchester Foundation Trust, Oxford Road, Manchester M13 9WL, UK; 3 arc Epidemiology Unit, School of Translational Medicine, The University of Manchester, Manchester M13 9PT, UK; 4 Department of Vascular Surgery, Central Manchester Foundation Trust, Oxford Road, Manchester M13 9WL, UK; 5 Department of Clinical Biochemistry, Central Manchester Foundation Trust, Oxford Road, Manchester M13 9WL, UK

## Abstract

**Background:**

Women with rheumatoid arthritis (RA) have increased morbidity and mortality due to coronary heart disease. Chronic systemic inflammation is known to accelerate atherosclerosis and increase arterial stiffness in patients, but other mechanisms may also be involved. Biomarkers of oxidant stress, inflammation, insulinaemia and endothelial dysfunction were measured in blood and urine from 46 RA patients and 48 age-matched controls. Plaque formation and intima-medial thickness (IMT) were measured using B-mode carotid Doppler scan.

**Findings:**

The prevalence of plaque was increased (p = 0.042) in RA patients between 50–59 years old compared to the same age group in controls. 8-isoprostane (p = 0.004), C-reactive protein (p < 0.001), interleukin-6 (p < 0.001), insulin (p = 0.035), adiponectin (p = 0.012), vascular cell adhesion molecule (VCAM) (p = 0.029) and E-selectin (p < 0.001) were all increased while selenium (p = 0.003) and LDL-cholesterol (p = 0.025) were both decreased in all RA patients. 8-isoprostane correlated with 10 year cardiac risk (r = 0.55, p < 0.001), VCAM with IMT (r = 0.37, p = 0.012) and E-selectin with rheumatoid factor titre (r = 0.43, p = 0.003) in RA patients. In the control group, age, carotid IMT, VCAM, systolic blood pressure and smoking status were all associated with plaque development whereas in RA patients only age was associated with plaque.

**Conclusion:**

The burden of atherosclerosis is particularly increased in middle-aged women with RA. Patients with RA have increased levels of oxidant stress, inflammation, insulin and soluble adhesion molecules. As the association between classical risk factors was much weaker in RA patients compared to controls, these additional factors may be more important in the accelerated development of atheroma in RA.

## Findings

Recent studies [1,2] have established that patients with rheumatoid arthritis (RA) have a 2-fold higher risk of myocardial infarction than the general population and, in those with RA for 10 years or more, the risk is increased to > 3-fold. The mechanisms behind this higher incidence of coronary heart disease in RA patients are not fully understood but may be related to vascular inflammation and vascular endothelial injury which is common in RA patients [3]. Newly diagnosed RA patients have significantly impaired endothelial function which can be improved with anti-inflammatory therapies [4]. Oxidant stress and the production of intracellular reactive oxygen species (ROS) have been implicated in the pathogenesis of a variety of cardiovascular diseases [5]. In synovial fluid in the inflamed rheumatoid joint, activated neutrophils produce large amounts of ROS and activated poly-morphonuclear cells are also likely to increase oxidant stress [6]. Hyperinsulinaemia is associated with increased long-term mortality following acute myocardial infarction in non-diabetic patients [7]; in vitro studies show that insulin has both atherogenic (in supraphysiological concentrations) and anti-atherogenic (in physiological concentrations) effects on the vessels. The latter vasodilatory action might be lost or down-regulated in the insulin resistant state, where increased insulin secretion in combination with hyperglycaemia leads to smooth muscle cell hypertrophy and hyperplasia, and excess synthesis of extra-cellular matrix proteins. Adiponectin, an adipose-specific secretory protein, has been implicated in insulin-resistance and development of atherosclerosis in obese and diabetic individuals; it has also been suggested that adiponectin has anti-inflammatory effects on the vascular wall [8]. Adhesion molecules are transmembrane glycoproteins that facilitate the recruitment of leucocytes to sites of inflammation including developing atherosclerotic lesions and circulating concentrations may be useful predictors of cardiovascular disease [9]. 

The present study was designed to examine the extent of oxidant stress, inflammation and hyperinsulinaemia in patients with RA and whether these factors may contribute to atherogenesis in this population. We also sought to determine the potential relationship between other, more classic, risk factors for atherosclerosis and subclinical atherosclerosis in RA.

## Methods

The study was approved by the Central Manchester Local Research Ethics Committee and all subjects gave written informed consent. We recruited 46 female RA patients and 48 age-matched female controls. Blood and urine samples were taken following an overnight fast and avoidance of alcohol for 48 hours. A detailed clinical history including family history of ischemic heart disease (FH of IHD) and current medications was taken. The 10-year risk of myocardial infarction was calculated. Examination included basic anthropomorphic measurements, blood pressure and, in RA patients, assessment of current disease activity using the 28-joint Disease Activity Score (DAS28).

Carotid Ultrasound was performed by a single operator using an ATL HDI 5000 scanner equipped with 7-4 MHz linear array transducer. IMT was reported as previously described [10]. Ultrasound images were also scored for plaque in the proximal common, distal common, carotid bulb, internal and external carotid arteries [11].

8-isoprostane (8-IP), malondialdehyde (MDA), selenium, total antioxidant capacity in whole and protein-free serum, vitamin A and vitamin E were assayed as previously described [12]. C-reactive protein (CRP) was assayed by sandwich ELISA technique using antibodies from Dakocytomation (Glostrop, Denmark). IL-6 was measured using a high-sensitivity ELISA kit (Diaclone, Besancon, France). Insulin was measured by radio-immunoassay [13], insulin sensitivity being calculated as HOMA-S = 1/(insulin × glucose/22.5). Adiponectin, VCAM, E-selectin and P- selectin were all measured using DuoSet^® ^ELISA development systems (R&D Systems, Minneapolis, MN).

Total cholesterol, HDL, TG and glucose were all determined by standard automated techniques and LDL-cholesterol estimated by the Friedewald formula. Rheumatoid factor was measured by nephelometry.

### Statistical Analysis

Values are shown either as medians with interquartile ranges or as the number of affected individuals with percentage of the total. Where appropriate, the Mann-Whitney U test or Fisher's Exact Probability test was used to calculate significance between groups. Significance of correlations between parameters was assessed by the Spearman Rank test. Univariate logistic regression analysis was conducted on candidate variables in the control and RA groups using the occurrence of plaque (0 or 1) as the response.

## Results

We studied 46 female RA patients with a median (IQR) age of 57 (51.5–60) years who had established RA disease for a median (IQR) of 12 (7–19) years. Of these patients, 33 (72%) were RF positive, 34 (74%) had radiographic erosions and 19 (41%) had at least one joint replacement. With regard to treatment, 44 (96%) had been treated with at least one disease-modifying anti-rheumatic drug (DMARD), 16 (35%) were currently taking steroids, 12 (26%) were using a non-selective NSAID and 11 (24%) were on a COX-2 inhibitor. The median (IQR) DAS28 score was 3.6 (2.4–4.6) indicating moderately active disease.

Risk factors for controls and RA patients are shown in Table 1 and, with the exception of calculated LDL-cholesterol which was lower in RA patients, there was no significant difference in other classic cardiovascular risk factors between groups. There was also no difference in the carotid IMT.

**Table 1:** Cardiovascular risk factors in RA patients and controls.

risk factor	Controls	RA patients	p value
age	57 (51.5–60)	56 (49–60)	ns
BMI	25.16 (23.30–28.75)	25.53 (23.39–29.55)	ns
waist/hip	0.818 (0.778–0.839)	0.846 (0.800–0.886)	ns
SBP	128 (117.5–143)	128 (122–140)	ns
DBP	78 (72–84)	80 (73.5–84.5)	ns
total cholesterol	5.75 (4.9–6.75)	5.5 (4.5–6.1)	ns
HDL-cholesterol	1.55 (1.30–2.03)	1.70 (1.40–2.00)	ns
triglycerides	1.20 (0.85–1.50)	1.10 (0.80–1.43)	ns
LDL-cholesterol	3.50 (2.73–4.33)	3.09 (2.34–3.78)	0.025
10 year cardiac risk	5.75 (3.20–10.35)	4.90 (1.58–7.93)	ns
IMT	0.055 (0.047–0.062)	0.053 (0.047–0.060)	ns
smoking	24 (50%)	30 (65%)	ns
hypertension	6 (13%)	12 (26%)	ns
FH of IHD	11 (23%)	16 (35%)	ns

Results are expressed either as median (interquartile range) or as the number of affected individuals (percentage of the total). Data were analysed using the Mann-Whitney U-test (continuous variables) or Fisher's Exact Probability test (dichotomous variables). ns = not significant. Units were expressed as follows: age as years; SBP and DBP as mmHg; total cholesterol, HDL-cholesterol, triglycerides and LDL-cholesterol as mmol/l; 10 year cardiac risk as %; IMT as cm.

Overall, there was no statistical difference between controls and RA patients with carotid plaque (Table 2). However, the prevalence of carotid plaque was significantly increased (p = 0.042) in RA patients aged 50–59 years old. In the < 50 year old age group, 2/13 patients but no controls had evidence of plaque formation. No difference in plaque formation was found in the older (> 60 years) age group.

**Table 2:** Frequency of Carotid Plaque with Age in RA patients and controls.

age (years)	controls	RA patients	p value
< 50	0/8 (0%)	2/13 (15%)	ns
50–59	6/26 (23%)	10/19 (53%)	0.042
> 60	8/14 (57%)	7/14 (50%)	ns
all subjects	14/48 (29%)	19/46 (41%)	ns

Values are shown as the number of affected individuals with percentage of the total. Fisher's Exact Probability test was used to calculate significance between groups.

ns = not significant.

**Figure 1:**
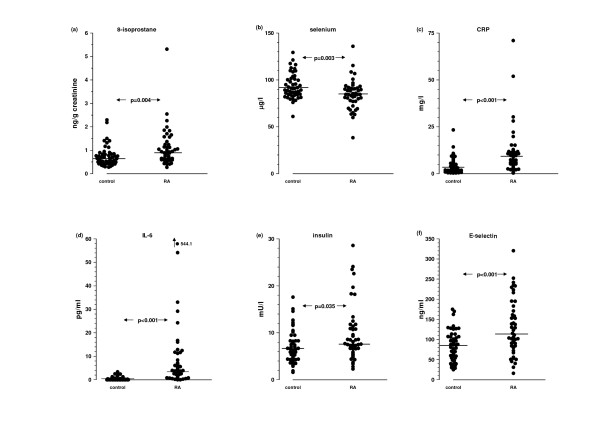
**Key markers of lipid peroxidation, antioxidant status, inflammation, insulinaemia and endothelial dysfunction in control and RA groups**. Medians are indicated. p values derived by the Mann-Whitney test.

The results for biomarkers are shown in Table 3 and Figure 1. In the RA group, we found clear evidence for increased lipid peroxidation as urinary 8-IP levels were significantly elevated (p = 0.004) compared to controls (Figure 1a). Plasma MDA, a less sensitive marker of lipid peroxidation, was unchanged. The total antioxidant capacity (in whole serum and protein-free serum), vitamin A and vitamin E levels were no different from control values. Serum selenium levels were significantly reduced (p = 0.003) in RA (Figure 1b). C-reactive protein (Figure 1c) and IL-6 (Figure 1d), were both significantly increased (p < 0.001) in RA. Similarly, insulin (Figure 1e) and adiponectin were also significantly increased (p = 0.035 and p = 0.012 respectively) while insulin sensitivity was decreased (p = 0.039) in RA patients. Plasma glucose levels were unchanged. Two of three soluble adhesion molecules, VCAM and E-selectin (Figure 1f), were increased (p = 0.029 and p < 0.001 respectively) in RA.

**Table 3:** Biomarkers of lipid peroxidation, antioxidant status, inflammation, insulinaemia and endothelial dysfunction in RA patients and controls.

marker	controls	RA patients	p value
8-IP	0.659 (0.449–0.845)	0.925 (0.598–1.248)	0.004
MDA	3.195 (2.825–3.434)	2.900 (2.648–3.346)	ns
selenium	91.14 (84.12–101.3)	84.55 (77.18–91.08)	0.003
TAC (ws)	730.0 (586.9–888.0)	728.2 (532.5–905.9)	ns
TAC (pfs)	343.7 (292.2–394.9)	334.7 (262.9–426.0)	ns
vitamin A	2.714 (2.472–3.191)	2.615 (2.241–3.224)	ns
vitamin E	13.63 (11.91–16.53)	13.41 (12.40–15.27)	ns
CRP	3.025 (1.269–5.246)	9.335 (5.201–11.79)	< 0.001
IL-6	0.046 (0–0.398)	3.723 (1.773–11.25)	< 0.001
insulin	6.65 (4.4–8.9)	7.65 (5.9–11.4)	0.035
glucose	4.700 (4.5–5.0)	4.700 (4.4–5.1)	ns
HOMA-S	0.718 (0.54–1.08)	0.615 (0.40–0.80)	0.039
adiponectin	3.369 (2.31–4.74)	4.543 (3.15–6.97)	0.012
VCAM	241.1 (200.4–260.8)	253.8 (224.7–298.0)	0.029
E-selectin	86.74 (54.19–107.3)	113.4 (86.74–113.4)	< 0.001
P-selectin	17.34 (14.49–23.62)	20.29 (16.47–25.40)	ns

Results are expressed as median (interquartile range). The Mann-Whitney U test was used to calculate significance between groups. ns = not significant. Units were expressed as follows: 8-IP as ng/g creatinine; MDA and vitamin A as μmol/l; selenium, VCAM, E-selectin and P-selectin as μg/l; TAC (ws and pfs) as mmol/l trolox equivalents; vitamin E, CRP and adiponectin as mg/l; IL-6 as ng/l; insulin as mU/l; glucose as mmol/l.

Spearman correlations between biomarkers and cardiovascular risk factors for controls and RA patients are shown in Table 4. In the RA group, urinary 8-IP correlated positively with age and smoking as well as with 10 year cardiac risk (r = 0.552, p < 0.001), E-selectin (r = 0.422, p = 0.004), TG (r = 0.392, p = 0.008) and CRP (r = 0.313, p = 0.034) and negatively with HDL (r = -0.418, p = 0.005). Serum selenium in RA was associated with HDL (r = 0.388, p = 0.009) and TG (r = -0.317, p = 0.036). As expected, IL-6 and CRP both correlated with DAS28 (r = 0.639, p < 0.001 and r = 0.316, p = 0.041 respectively). Insulin correlated positively with BMI, SBP and glucose (r = 0.314, p = 0.034) and negatively with adiponectin (r = -0.432, p = 0.003). VCAM was associated with BMI while E-selectin was associated with BMI, LDL and smoking as well as RF titre (r = 0.429, p = 0.003). We found that VCAM was associated with carotid IMT in both patients and controls. In the patient group, we also found a negative correlation between IL-6 and IMT.

**Table 4:** Spearman correlations between biomarkers and potential cardiovascular risk factors.

	cardiovascular risk factor					
	
biomarker	age	BMI	LDL	IMT	SBP	smoking
controls:						
8-IP	0.090	0.272	0.039	0.131	0.161	0.416**
selenium	0.299*	-0.305*	0.227	-0.031	-0.240	-0.281
CRP	0.059	0.427**	0.238	0.205	0.255	0.043
IL-6	0.091	0.421**	0.018	0.201	0.248	0.214
insulin	0.009	0.408**	0.148	0.125	-0.030	0.014
adiponectin	0.142	-0.256	0.015	0.065	0.132	0.281
VCAM	0.413**	0.062	0.327*	0.339*	0.191	0.232
E-selectin	0.212	0.111	0.434**	0.257	0.252	0.188

RA patients:						
8-IP	0.321*	0.198	0.271	0.170	0.088	0.308*
selenium	0.160	0.100	0.189	0.073	0.191	0.117
CRP	0.221	0.160	0.019	0.184	0.215	0.257
IL-6	-0.185	-0.251	-0.234	-0.342*	-0.242	0.049
insulin	0.245	0.594**	0.119	0.254	0.369*	0.117
adiponectin	0.265	-0.331*	-0.049	0.067	0.127	-0.031
VCAM	0.102	0.297*	0.214	0.369*	0.188	0.019
E-selectin	0.015	0.389**	0.388**	0.198	0.135	0.372*

Results are expressed as r values. * p < 0.05; ** p < 0.01.

With regard to association with carotid plaque (Table 5), univariate logistic regression analysis of the control group found that plaque was associated with age (p = 0.013), IMT (p = 0.008), VCAM (p = 0.007), SBP (p = 0.014) and smoking (p = 0.010). In contrast, in the RA group, only age (p = 0.034) was significantly associated with plaque.

**Table 5:** Univariate logistic regression of factors associated with carotid plaque in RA patients and controls

	controls			RA patients		
variable	odds ratio	ROC	p value	odds ratio	ROC	p value
age	1.22 (1.04–1.42)	0.79	0.013	1.11 (1.01–1.22)	0.68	0.034
IMT	2.79 (1.32–5.91)	0.78	0.008	1.34 (0.77–2.36)	0.59	0.300
VCAM	1.02 (1.01–1.04)	0.77	0.007	1.00 (0.99–1.01)	0.62	0.375
SBP	1.05 (1.01–1.10)	0.76	0.014	1.04 (0.99–1.08)	0.61	0.063
smoking	1.09 (1.02–1.17)	0.74	0.010	1.05 (0.99–1.11)	0.61	0.109
IL-6	1.74 (0.88–3.43)	0.71	0.110	0.97 (0.91–1.04)	0.52	0.449
E-selectin	1.02 (1.00–1.04)	0.71	0.055	1.00 (0.99–1.01)	0.49	0.809
8-IP	3.83 (0.86–17.0)	0.69	0.077	1.01 (0.49–2.08)	0.48	0.972
LDL	1.76 (0.92–3.34)	0.67	0.086	1.34 (0.68–2.66)	0.56	0.394
adiponectin	1.27 (0.98–1.65)	0.66	0.066	1.08 (0.88–1.33)	0.62	0.436
insulin	1.17 (0.98–1.40)	0.63	0.088	1.00 (0.91–1.10)	0.50	0.980
selenium	0.98 (0.93–1.03)	0.61	0.365	0.98 (0.94–1.02)	0.58	0.296
BMI	1.05 (0.96–1.15)	0.58	0.319	0.95 (0.85–1.07)	0.54	0.431
CRP	1.07 (0.93–1.23)	0.56	0.358	1.00 (0.96–1.05)	0.53	0.884

Odds ratio (95% confidence interval), area under the receiver operating characteristic (ROC) curve and p value using a univariate logistic regression model with occurrence of carotid plaque (0 or 1) in subjects as the response.

## Discussion

The causes of increased coronary heart disease and cardiovascular mortality in RA patients may be multi-factorial, but cannot be explained merely by conventional risk factors such as age, gender, hyperlipidaemia, hypertension, smoking or excess weight [14].

It is still a matter of debate [5] whether the increased oxidant stress present in cardiovascular disease has a primary causative role or is rather a vascular sequel of disease progression. In the atherosclerotic process, lipids are the first line of radical attack and raised levels of 8-IP have been found in both serum and synovial fluid from patients with different rheumatic diseases including a small number with RA [15]. We have shown increased urinary 8-IP levels in RA patients, the median level being 40% greater than control. In addition, urinary 8-IP had a strong and significant association with 10 year cardiac risk in our RA group. Elevated levels of MDA, a product of polyunsaturated fat oxidation, have been reported in the serum and synovial fluid of patients with RA [6] but we found no difference in plasma MDA levels between groups. Selenium was the only measure of antioxidant capacity that showed a significant reduction in our RA group in agreement with an earlier finding [16]. Long-term selenium supplementation in RA patients restored selenium and glutathione peroxidase levels in serum and red blood cells, but failed to improve glutathione peroxidase levels in granulocytes [17].

Pro-inflammatory cytokines are involved in the development of RA and mediate synovial inflammation in the rheumatoid joint. Elevated IL-6 levels have been found in synovial fluid and serum in RA patients [18] and serum IL-6 levels correlated with disease activity and radiographic joint damage [19]. CRP is produced in the liver in response to IL-6 and, in addition to its role as a marker of disease activity in RA, CRP has also been shown to be associated with carotid plaque in RA [20]. As anticipated, both IL-6 and CRP levels were increased in our patient group and correlated with the DAS28; however, neither marker was associated with plaque. Of course, it should also be borne in mind that plaque formation is the result of exposure to a range of known and unknown risk factors over the whole of a patient's lifetime.

Non-diabetic patients with hyperinsulinaemia tend to be more obese and suffer more frequently from hypertension and myocardial infarction [7]. Insulin was elevated and HOMA-S decreased in our RA patients, but glucose levels were no different from controls. Serum adiponectin was also increased in our RA patients. Adiponectin is known to suppress the expression of adhesion molecules in vascular endothelial cells and cytokine production from macrophages, thus inhibiting the inflammatory processes that occur during the early phases of atherosclerosis. Increased adiponectin levels in RA may therefore be in part an attempt to suppress a profound inflammatory insult [21]. The decrease in total LDL-cholesterol in our RA group may imply protection from atheroscelerosis. It has been shown [22] that individuals with higher adiponectin levels had lower LDL-cholesterol levels and that, when adiponectin was raised, insulin resistance was low and the lipid profile good.

We found that two biomarkers of endothelial activation, E-selectin and VCAM, were raised in RA patients supporting the notion that patients with vascular changes may be most prone to atherosclerotic complications. VCAM is associated with IMT in patients and controls; E-selectin with RF titre in our patients. In the Norfolk Arthritis Registry, Goodson [14] noted that the risk of cardiovascular death was increased in patients with rheumatoid factor.

Carotid plaque develops earlier in our RA group, the incidence being significantly higher in patients between 50–59 years than in the same control age group. In previous studies [23,24], carotid plaques tended to have a higher frequency in RA patients without reaching statistical significance. The intima-media thickness of the common carotid artery measured by ultrasound imaging has been shown to be a reliable marker of preclinical atherosclerosis [25], but no difference was found between our groups. However, the great majority of our patients had been treated with DMARDs and methotrexate, for example, is known to decrease IMT [26]. Univariate logistic regression analysis showed that only age independently predicted plaque formation in the RA group. Plaque rupture is now acknowledged as the major cause of unstable angina, myocardial infarction and sudden cardiac death; strategies to reduce this risk include inhibition of inflammatory cytokines and the use of antioxidants [27]. We conclude that the increase in oxidant stress, inflammation, insulin resistance and adhesion molecules demonstrated in our RA patients could all exert additive atherogenic effects.

## Competing interests

The authors declare that they have no competing interests.

## Authors' contributions

PWP assayed most of the biomarkers, carried out the statistical analysis and prepared the manuscript. YA contributed to study design, data collection and analysis. HB was involved in carotid ultrasound and data collection. DL contributed to study design and data collection. SLH was involved in data collection and approval of the manuscript. APY performed sample processing and insulin assay. MGW carried out Vascular Laboratory supervision and approved the manuscript. IL coordinated the study and participated in discussions about insulin sensitivity. INB conceived and designed the study, and was involved in drafting the manuscript. All authors have read and approved the final manuscript.
